# New Insights From Transcriptomic Data Reveal Differential Effects of CO_2_ Acidification Stress on Photosynthesis of an Endosymbiotic Dinoflagellate *in hospite*

**DOI:** 10.3389/fmicb.2021.666510

**Published:** 2021-07-19

**Authors:** Marcela Herrera, Yi Jin Liew, Alexander Venn, Eric Tambutté, Didier Zoccola, Sylvie Tambutté, Guoxin Cui, Manuel Aranda

**Affiliations:** ^1^Red Sea Research Center (RSRC), Biological and Environmental Sciences and Engineering Division (BESE), King Abdullah University of Science and Technology (KAUST), Thuwal, Saudi Arabia; ^2^Marine Department, Centre Scientifique de Monaco, Monaco, Monaco

**Keywords:** carbon-concentrating mechanism, coral-dinoflagellate symbiosis, ocean acidification, Symbiodiniaceae, *F’/Fm’*, *p*CO_2_, gene expression

## Abstract

Ocean acidification (OA) has both detrimental as well as beneficial effects on marine life; it negatively affects calcifiers while enhancing the productivity of photosynthetic organisms. To date, many studies have focused on the impacts of OA on calcification in reef-building corals, a process particularly susceptible to acidification. However, little is known about the effects of OA on their photosynthetic algal partners, with some studies suggesting potential benefits for symbiont productivity. Here, we investigated the transcriptomic response of the endosymbiont *Symbiodinium microadriaticum* (CCMP2467) in the Red Sea coral *Stylophora pistillata* subjected to different long-term (2 years) OA treatments (pH 8.0, 7.8, 7.4, 7.2). Transcriptomic analyses revealed that symbionts from corals under lower pH treatments responded to acidification by increasing the expression of genes related to photosynthesis and carbon-concentrating mechanisms. These processes were mostly up-regulated and associated metabolic pathways were significantly enriched, suggesting an overall positive effect of OA on the expression of photosynthesis-related genes. To test this conclusion on a physiological level, we analyzed the symbiont’s photochemical performance across treatments. However, in contrast to the beneficial effects suggested by the observed gene expression changes, we found significant impairment of photosynthesis with increasing *p*CO_2_. Collectively, our data suggest that over-expression of photosynthesis-related genes is not a beneficial effect of OA but rather an acclimation response of the holobiont to different water chemistries. Our study highlights the complex effects of ocean acidification on these symbiotic organisms and the role of the host in determining symbiont productivity and performance.

## Introduction

Rising levels of anthropogenic carbon dioxide (CO_2_) are transforming the chemistry of our oceans; the average seawater pH has already decreased by 0.1 pH units (equivalent to almost 30% increase in acidity) since the Industrial Revolution and it is predicted to drop even further by the end of this century (Magnan et al., 2016; Hoegh-Guldberg et al., 2017). Thus, there is a great concern regarding the future of marine ecosystems and the ecological services they provide; particularly for coral reefs, as these depend entirely on the persistence of corals and other calcifiers (Hoegh-Guldberg et al., 2007, 2017; Pandolfi et al., 2011; Hughes et al., 2017). As CO_2_ dissolves in water, carbonic acid (H_2_CO_3_) is formed and protons (H^+^) are released, which lowers the pH (i.e., more acidic) but also reacts with carbonate ions (CO_3_^2–^), which in turn reduce carbonate concentrations and saturation state (Ω). Calcifying organisms are then unable to build skeletons, have slower growth and are more sensitive to disturbances (Doney et al., 2009).

Much attention has been focused on the detrimental effect of ocean acidification (OA) on calcification (Orr et al., 2005; Hofmann et al., 2010; Andersson and Gledhill, 2013), yet, it is unclear how it affects other physiological processes (Kroeker et al., 2010, 2013). Specifically, there is a growing interest in understanding how acidification will impact photosynthetic organisms (Mackey et al., 2015; Connell et al., 2018). For example, changes in diversity, structure, and productivity of phytoplankton communities are predicted to have profound consequences for marine food webs and biogeochemical processes (Dutkiewicz et al., 2015). Indeed, increased biomass of primary producers, such as sea grasses and algae, has been shown to alter the ecological functioning of benthic ecosystems by favoring the proliferation of some species and demise of others (Hall-Spencer et al., 2008; Connell et al., 2017). For symbiotic corals, OA may even have a greater impact on bleaching and productivity than on calcification (Anthony et al., 2008). Nonetheless, the effects are multi-faceted and complex. Like other aquatic photosynthetic organisms, the endosymbionts of corals (of the family Symbiodiniaceae) also have active CO_2_-concentrating mechanisms (CCMs) (Al-Moghrabi et al., 1996; Leggat et al., 1999) that allow them to cope with the challenges of living in a carbon-limited environment (Moroney and Ynalvez, 2007). To fuel photosynthesis, CO_2_ must be converted to HCO_3_^–^ (and vice versa) so it can be transported inside the cell and ultimately reach RuBisCO, the enzyme that catalyzes the first step of CO_2_ fixation. However, *in hospite*, this process is actively regulated by the host (Barott et al., 2015) through proteins like carbonic anhydrases and bicarbonate transporters that catalyze the interconversion of CO2 and bicarbonate and mediate their transport. Certainly, this implies that the symbiont relies on the host to provide a suitable environment that supports its functioning (e.g., *in hospite* nutrient availability) so that if the physiological response of the latter is compromised, that of the symbiont will be too. Thus, even if productivity of the holobiont increases under elevated *p*CO_2_ (Strahl et al., 2015; Biscéré et al., 2019), its performance and survival are ultimately limited by the physiological capabilities of the coral, which can either be negatively impacted (e.g., reduced metabolism, increased oxidative stress, apoptosis, etc.) (Kaniewska et al., 2012) or (seemingly) unaffected (Wall et al., 2014; Tambutté et al., 2015; Davies et al., 2018).

Similar to the specific stress sensitivity of their cnidarian hosts (Anthony et al., 2008; Crawley et al., 2010; Edmunds, 2012; Kaniewska et al., 2012; Towanda and Thuesen, 2012; Jarrold et al., 2013; Gibbin and Davy, 2014; Hoadley et al., 2015b; Klein et al., 2017; Davies et al., 2018), differential responses to OA have also been observed among Symbiodiniaceae types (Buxton et al., 2009; Brading et al., 2011). This is not surprising considering the remarkable intra- and inter- specific functional diversity (LaJeunesse et al., 2018), including species-specific CCMs (Brading et al., 2013). For example, a recent study (Aranda et al., 2016) showed substantial differences in the number of bicarbonate transporters in the genomes of coral-associated endosymbionts; in particular, *Symbiodinium microadriaticum* appears to have significantly more compared to *Breviolum minutum*, which likely has fundamental implications for carbon acquisition and (indirectly) productivity. Various combinations of host and symbiont genotypes may provide physiological (dis)advantages under changing ocean conditions. Hence, examining the response of different symbiotic associations to elevated *p*CO_2_ can provide valuable understanding of OA effects on corals (Hoadley et al., 2015a).

Here, we investigated the global transcriptomic response of the endosymbiont *Symbiodinium microadriaticum* of the Red Sea coral *Stylophora pistillata* (Esper 1797) to long-term seawater acidification stress. This pocilloporid coral is not only one of the most abundant species and major contributor to reef structures in the Indo-Pacific (Veron, 2000) but has also been used as a model organism to study ecological, physiological, and evolutionary aspects of the cnidarian-dinoflagellate symbiosis (Ferrier-Pagès et al., 2003; Franklin et al., 2004; Putnam et al., 2008; Venn et al., 2013; Vidal-Dupiol et al., 2013; Maor-Landaw et al., 2014; Cohen and Dubinsky, 2015; Tambutté et al., 2015; Zoccola et al., 2015; Voolstra et al., 2017; Liew et al., 2018). Colonies of this coral have been successfully cultured under CO_2_-driven low pH conditions for almost 10 years, thus providing a unique opportunity to examine the effects of chronic exposure to high CO_2_. Further, most studies on OA represent data from short-term experiments that range from days to few weeks and focused on the physiological impact of coral calcification (Kaniewska et al., 2012; Chan and Connolly, 2013), however, little is known for the algal partner, especially with regard to responses on the molecular level. This study presents novel observations on the responses of a common coral symbiont to acidification stress.

## Materials and Methods

### Experimental Incubations

**FIGURE 1:**
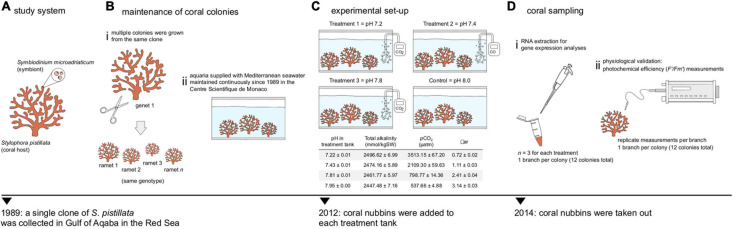
Schematic of the experimental design. **(A)** A single clone of the Red Sea coral *Stylophora pistillata* (symbiotic with *Symbiodinium microadriaticum* CCMP2467) was collected in 1989. **(B)** The same coral colony was **(i)** fragmented into multiple nubbins and **(ii)** maintained in the aquaria system of the Centre Scientifique de Monaco. **(C)** At least 3 nubbins were added to different pH treatments in 2012: pHs 7.2, 7.4, 7.8 and a control pH 8.0. Details on carbonate chemistry are shown as means ± SD; Ωar, saturation state of aragonite [taken from Liew et al. (2018)]. **(D)** Coral nubbins were retrieved in 2014 after being subjected to their respective treatments for 2 years. **(i)** RNA extractions were performed and **(ii)** photochemical efficiency (*F’/Fm’*) were measured to analyze changes in gene expression and assess the photo-physiological status, respectively.

Multiple colonies of a single clone of *S. pistillata* were subjected to long-term seawater acidification in an experimental setup at the Centre Scientifique de Monaco that has been maintained continuously from the early 2010s (Venn et al., 2013; Tambutté et al., 2015; Liew et al., 2018). Based on nuclear ITS and mitochondrial COI, colonies were previously typed to be *S. pistillata* clade 4 (Voolstra et al., 2017), which is found throughout the northwest Indian Ocean including the Red Sea, the Persia/Arabian Gulf and Kenya (Keshavmurthy et al., 2013). Coral fragments were kept simultaneously in four aquaria, each supplied with Mediterranean seawater with an exchange rate of 70% per hour, salinity of 38 ppt, temperature of 25°C and irradiance of 170 μmol photons/m^2^s white light on a 12:12 h light:dark photoperiod provided by HQI-10000K metal halide lamps (BLV Nepturion), and fed with freshly hatched *Artemia* brine shrimps twice per week. Carbonate chemistry was manipulated by bubbling CO_2_ to reduce pH to the reach values of pH 7.2, an extremely low value used to generate and study new phenotypes (Tambutté et al., 2015; Liew et al., 2018), pH 7.4, which represent extreme values observed today in some environments like volcanic CO_2_ vents where mean pH can range between 7.4 and 7.6 (Hall-Spencer et al., 2008) and pH 7.8 or near future natural conditions as projected by the IPCC scenario RCP8.5 (Magnan et al., 2016), while a control aquarium was maintained at the current average seawater pH 8.0 (Doney et al., 2009). pH and temperature were constantly checked with a custom-made monitoring system (Enoleo, Monaco) so that similar conditions prevailed in each aquarium except for the carbonate chemistry. Details on chemistry parameters, pH and alkalinity measurements, and aquarium maintenance are provided in Tambutté et al. (2015) and Liew et al. (2018). For this experiment (same as Liew et al., 2018), coral fragments (three per tank) were added to the system in 2012 and taken out in 2014 (at the same time) after being subjected to the respective treatments for 2 years (Figure 1).

### Identification of Differentially Expressed Genes

High-quality total RNA was extracted from three replicate coral nubbins (one per colony, three colonies in each tank) that were kept in all different conditions for 2 years. Twelve strand-specific mRNA libraries were generated using the NEB Next Ultra Directional RNA Library Prep Kit for Illumina (New England Biolabs) and sequenced in six lanes of the HiSeq 2000 platform (Illumina) to retrieve a total of 674 million paired-end reads (101 bp). RNA-seq data was analyzed as implemented in Kallisto v0.44.0 (Bray et al., 2016) and its companion tool Sleuth v0.28.0 (Pimentel et al., 2017). Kallisto pseudo-aligns reads to a reference to produce a list of transcripts compatible with each read while avoiding alignment of individual bases, thus achieving much faster results compared to other approaches. Details on pseudo-alignment statistics are provided in Supplementary Table 1. Quantifications of gene expression in transcripts per million reads (TPM) resulting from the bootstrapping performed in Kallisto were then analyzed with Sleuth. Briefly, Sleuth builds a response error model that allows for the decoupling of biological variance from inferential variance to ultimately identify differentially expressed genes (DEGs). Significance of the differential expression levels is evaluated through a likelihood test (LRT), after which Sleuth returns a *q*-value per coding sequence (i.e., the corrected *p*-value following the Benjamini-Hochberg correction for reducing the false discovery rate (FDR) due to multiple testing). Here, we chose a threshold of 0.05 for the *q*-value (corresponding to an FDR of 0.05), below which we considered the DEGs to be significant. Differential expression levels are outputted as a “beta” (*b*) value, which in order to get a differential expression level index similar to the classically used log2 fold-change, we transformed by raising the number *e* to the b-th power (i.e., *e*^*b*^).

While Liew et al. (2018) analyzed the transcriptomic response of *S. pistillata*, that study solely focused on the differential expression of methylated genes in the host, particularly those related to growth and biomineralization pathways. Thus, here we assessed the overall response of *S. pistillata*, along with the response of its endosymbiont *S. microadriaticum*. All analyses described above were carried out for both data sets. Reads were mapped to *S. microadriaticum* (Aranda et al., 2016) and *S. pistillata* (Voolstra et al., 2017) gene models (reference genomes can be found at^1^), and DEGs were identified by contrasting samples from all experimental conditions (pHs 7.2, 7.4, and 7.8) against the control (pH 8.0). Normalized expression values were log (*x* + 1) transformed and principal component analyses (based on Euclidean distances) were carried out after to assess the relationship between samples. Differences between treatments were tested using the function “Adonis” as implemented in the R (v3.5.1) (R Core Team, 2018) package “vegan” (Oksanen et al., 2019).

### Functional Enrichment Analyses

GO term enrichment analyses were performed with topGO (Alexa et al., 2006) using a self-developed R script^2^ as described in Liew et al. (2018). Only GO terms with *p* < 0.05 and occurring at least five times were considered enriched. Furthermore, KEGG Orthology (KO) annotations were merged from the KEGG Automatic Annotation Server^3^ (Moriya et al., 2007) (with the parameters ‘‘GHOSTZ,’’ ‘‘eukaryotes’’ and ‘‘bi-directional best hit’’) and the results of the gene models of both *S. microadriaticum* and *S. pistillata*^4^. KEGG pathway enrichment analysis of DEGs were carried out using Fisher’s exact test and subsequent multiple testing correction via false discovery rate (FDR) estimation. Only pathways with *p* < 0.05 were considered significant. The R package “GOplot” (Walter et al., 2015) was used to visualize the relationship between genes and selected functional categories. This is presented as a circular plot where the inner ring shows the z-score and the outer ring scatterplots of the expression levels for the genes assigned to each GO term. The z-score is calculated as the number of up-regulated genes minus the number of down-regulated genes divided by the square root of the count (Walter et al., 2015).

### Symbiont “Typing”

In order to identify the main symbiont of the *S. pistillata* colonies kept in the aquaria system at the Centre Scientifique de Monaco, RNA-seq data was mapped to SymPortal’s (Hume et al., 2019) reference database. Briefly, SymPortal is a platform for phylogenetically resolving Symbiodiniaceae taxa using ITS2 rDNA amplicon data. Its reference database contains thousands of ITS2 sequences from taxa belonging to the seven named genera (formerly genus *Symbiodinium* containing “clades A–I”). Read counts were calculated using Kallisto and based on this, A1 (>97.5%) was determined to be the most abundant sequence in all samples (Supplementary Figure 1). Symbiodiniaceae taxa associated with a dominant ITS2 designated sequence A1 is, in turn, associated to the species definition of *S. microadriaticum* (LaJeunesse, 2001). Yet, it is noteworthy clarifying that, here, the binomial *S. microadriaticum* refers to the strain CCMP2467, which was originally isolated from *S. pistillata* from the Gulf of Aqaba and was the same symbiont strain used to sequence the genome (Aranda et al., 2016).

### Physiological Validation: Photochemical Efficiency Measurements

Light-acclimated yields (*F’/Fm’*) were recorded in triplicate (one branch per colony and multiple measurements were taken for each branch at the same distance) with a Pulse Amplitude Modulated fluorometer (Junior-PAM, Walz, Germany) to assess the photo-physiological status of colonies at the different pH treatments (see raw data in Supplementary Table 2). Although measurements were not taken from the exact same fragments from which RNA was extracted, all colonies originated from the same coral genet (Figure 1) and phenotypes have been validated multiple times. A one-way analysis of variance (ANOVA) as implemented in R was conducted to assess the effect of pH in symbiont’s photochemical efficiency. Differences were further identified through Student-Newman-Keuls (SNK) *post hoc* tests.

## Results

Here, we investigated the *in hospite* transcriptomic response of *S. microadriaticum* in the Red Sea coral *S. pistillata* under long-term (2 years) acidification stress. Based on previous evidence showing beneficial effects from elevated *p*CO_2_ on productivity (Strahl et al., 2015; Biscéré et al., 2019), we focused on genes and processes involved in photosynthesis and carbon acquisition, which were also enriched in our differential gene expression analysis. Furthermore, we integrated the gene expression changes in the symbiont and host to assess the effect of low pH on both partners and overall response of the holobiont. Finally, we tested the conclusions from our transcriptomic analyses on the physiological level by measuring symbiont’s light-acclimated photochemical yields and interpreting relevant molecular responses of the host.

### Differential Gene Expression Following Chronic Exposure to High *p*CO_2_

**FIGURE 2:**
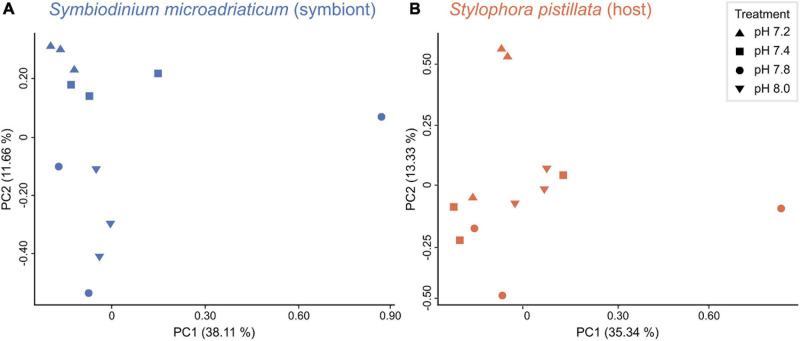
Relationship between samples. Principal component analysis (PCA) of **(A)** *in hospite Symbiodinium microadriaticum* CCMP2467 and **(B)** *Stylophora pistillata* samples across all four treatments.

**FIGURE 3:**
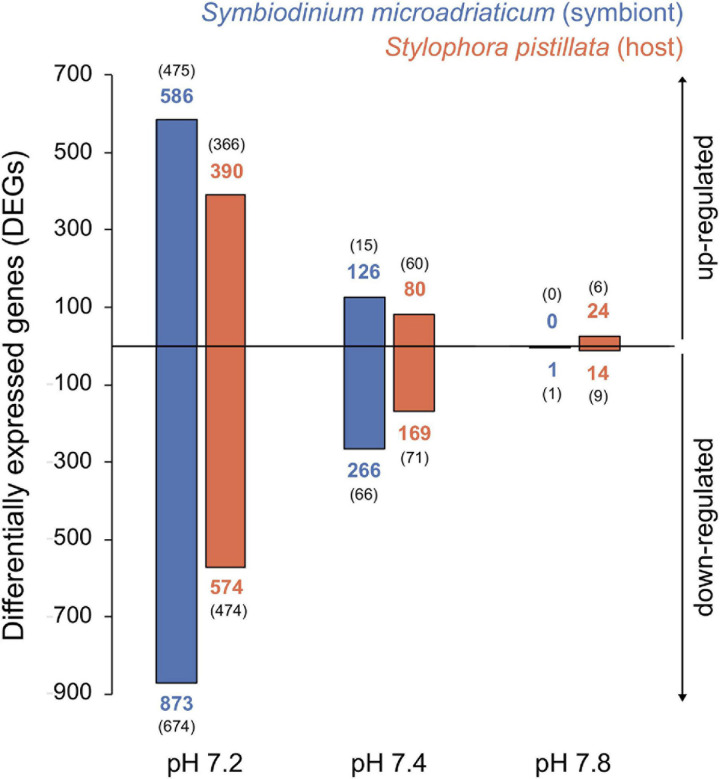
Barplot showing number of differentially expressed genes (DEGs) for the symbiont (blue) and coral host (orange) across pH treatments compared to the control. Numbers in bold indicate the total number of DEGs for each category whilst numbers in parenthesis show unique genes (exclusive to that treatment). Top and bottom panels depict direction of the change, up- and down-regulation, respectively.

Principal component analyses revealed that symbiont samples clustered according to treatment (Figure 2A), yet PERMANOVA tests did not show significant differences in gene expression between treatments (*p* = 0.08). The opposite was true for the host (Figure 2B, PERMANOVA *p* = 0.01), although no significant differences were observed among treatments (*post hoc* tests *p* > 0.05). Overall, gene expression patterns revealed a weaker transcriptomic response (that is, a lower number of DEGs) to the experimental treatments for the symbiont (1,230 unique genes representing 2.50% of the transcriptome) compared to the coral host (986 unique genes corresponding to 3.80%). As expected, more differentially expressed genes (DEGs) were identified in response to the strongest treatment of pH 7.2 for both partners, followed by at least three times less DEGs in the other conditions. A total of 1,459 (964), 392 (249), and 1 (38) DEGs were identified for the symbiont (host) in pH 7.2, 7.4, and 7.8, respectively (Figure 3). In regard to *S. microadriaticum* CCMP2467 approximately 45% of the DEGs had ≥ 2-fold change in expression (all of them up-regulated) and, again, most were observed in pH 7.2 (Figure 3). Only one DEG was identified at pH 7.8 (Smic24339), and it encoded for dimethylglycine dehydrogenase, a mitochondrial enzyme involved in pathways of degradation of amino acids and was only found in this treatment. Further, comparison of gene expression changes between pH 7.2 and 7.4 identified 310 shared DEGs, from which 111 and 199 were up- and down-regulated, respectively. Directionality of these changes was consistent across both treatments, although the magnitude of change was not. That is, 160 genes showed a higher fold change in expression in the pH 7.2 treatment whilst the remaining 150 had a lower expression compared to pH 7.4 (Supplementary Table 3). Patterns in expression for the *S. pistillata*, on the other hand (Figure 3), were similar to the symbiont’s, with most genes being down-regulated (at least in pH treatments 7.2 and 7.4).

### Impact of Low pH on Specific Biological Functions: Enrichment of Genes Related to Photosynthesis and Carbon Acquisition Processes

We conducted GO and KEGG pathway enrichment analyses to further assess the functional impact of the previously identified DEGs. A total of 114 significantly enriched GO terms across all treatments were identified for *S. microadriaticum* CCMP2467 (81, 26, and 7 from pH 7.2, 7.4, and 7.8, respectively, see Supplementary Tables 4A–C). Particularly noteworthy terms (most of them underrepresented) were related to translation and transcription, regulation of metabolic processes, and cellular components (ribosome, cytoplasm and microtubule complexes, cilium, among others). We also identified a few DEGs (with ≥ 2-fold change, all of them up-regulated) encoding for ion-transport activity; yet the associated GO terms were not significantly enriched.

**FIGURE 4:**
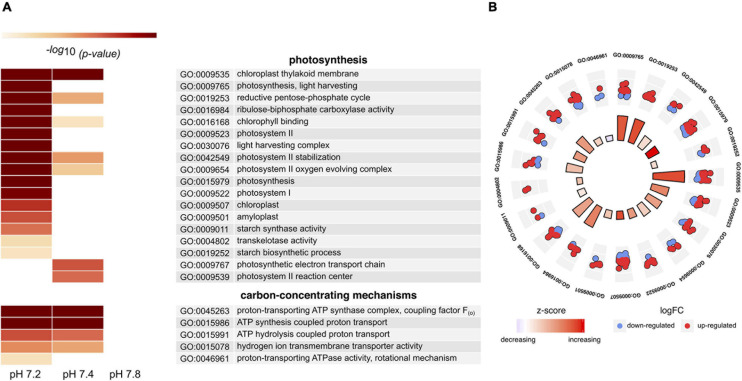
Differential transcriptomic response of *in hospite Symbiodinium microadriaticum* CCMP2467 to CO_2_ acidification stress. **(A)** Heat map showing GO terms related to photosynthesis and carbon concentrating mechanisms. Empty boxes denote differences that were not significant (*p* ≥ 0.05). Annotation of each term is described in the table and *p*-values are provided in Supplementary Table 8. **(B)** Circular plot showing selected GO terms enriched in pH 7.2. Statistical significance (log_10_ adjusted *p*-value) of each GO term is shown by the height of the bars in the inner circle, while the color represents the overall regulation effect of each process as indicated by the z-score (red – increased, white – unchanged, blue – decreased). The outer circle scatterplots show the differentially expressed genes assigned to each process, where red and blue represent genes that are up- and down-regulation, respectively.

We further focused on terms related to photosynthesis and carbon acquisition mechanisms as these have been previously reported to potentially benefit from increased CO_2_. Due to the lack of DEGs, we did not find any enriched GO terms for the pH 7.8 treatment, whilst at least 23 were identified for the other two conditions (Figure 4A, Supplementary Tables 4A–C). From these, most of them were predominant in pH 7.2 (21 compared to only 11 in pH 7.4) and related to photosynthesis light-harvesting proteins, the Calvin cycle and relevant enzymes (RuBisCO, transketolase and ferredoxin-NADP-reductase), as well as various cellular components and processes associated to photosystems I and II complexes. Fewer terms were associated with carbon acquisition mechanisms; most of them linked to genes encoding different subunits of the ATP synthase motor. Since pH 7.2 had the largest number of enriched GO terms, we investigated the expression changes of the genes associated with the corresponding processes (Figure 4B). A total of 172 DEGs were involved in these functional categories with most genes being over-expressed, thus suggesting an overall up-regulation of the process. Further, two of four significantly enriched KEGG pathways (Supplementary Table 5) in this treatment were related to photosynthesis (map00195) and carbon acquisition in photosynthetic organisms (map00710). DEGs involved in these pathways were mostly up-regulated with ≥ 2-fold increase in expression (Supplementary Table 6).

**FIGURE 5:**
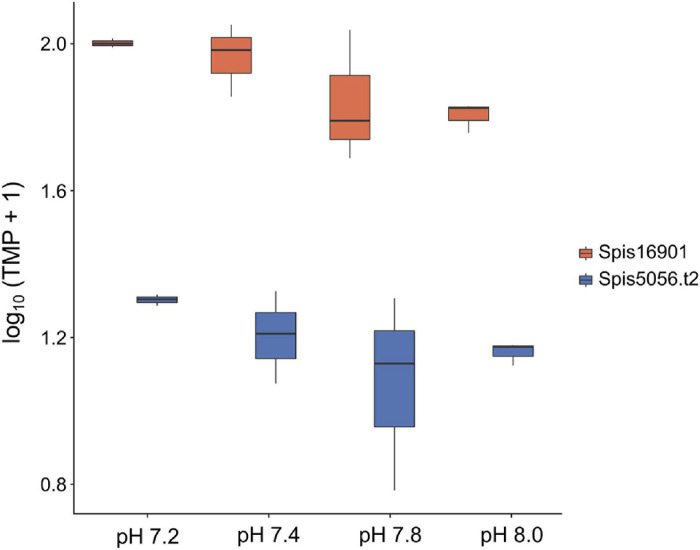
Absolute gene expression (transcripts per million) of two *Stylophora pistillata* genes encoding for bicarbonate transporter-like proteins across pH treatments. Although expression of these genes was not significantly different among conditions (ANOVA_*Spis*__16901_ *F* = 3.023, *p* = 0.094, ANOVA_*Spis*__5056__.t__2_ *F* = 1.402, *p* = 0.311), it followed the order pH 7.2 > 7.4 > 7.8 > 8.0.

To complement the analyses on the symbiont, we also performed a GO enrichment analyses for the transcriptional response of the host *S. pistillata*. This analysis revealed an overall enrichment of metabolic functions, RNA translational and transcriptional mechanisms, stress responses and cellular components (see Supplementary Tables 7A–C). Echoing the findings in the symbiont we also found more terms significantly enriched in the pH 7.2 treatment (167) compared to pH 7.4 (82) and pH 7.8 (94). Particularly relevant were terms related to ion activity and calcification across all treatments, but bicarbonate transport (GO:0015701) stood out (though it was only present in pH 7.2). We identified two genes (Spis16901 and Spis5056.t2) encoding for bicarbonate transporter like proteins, and these were significantly up-regulated (≥ 2-fold change). Although not significant (ANOVA_*Spis*__16901_ *F* = 3.023, *p* = 0.094, ANOVA_*Spis*__5056__.t__2_ *F* = 1.402, *p* = 0.311), the absolute expression (TPM) of these genes followed the order pH 7.2 > 7.4 > 7.8 > 8.0 (Figure 5). Also interesting was the term symbiont-containing vacuole membrane (GO:0020005) present in pH 7.8, which was associated to Spis21140. This gene, which annotates for an interferon-induced guanylate-binding protein 2, was significantly over-expressed and had a 2.5-fold change in expression.

### Physiological Response to Acidification Stress

**FIGURE 6:**
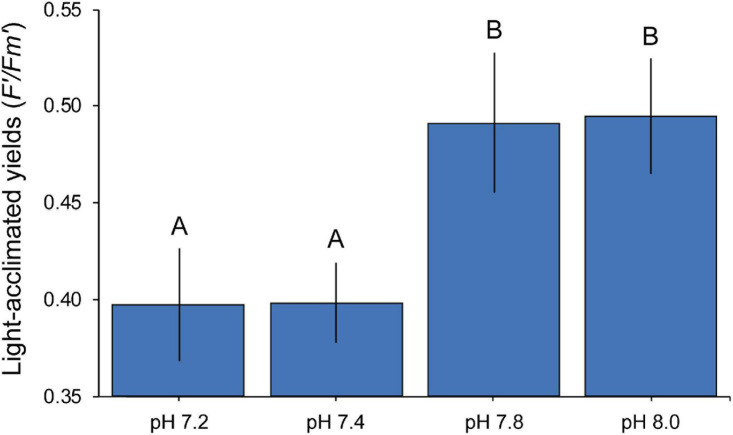
Mean (± 1 SE) photochemical efficiencies of *Symbiodinium microadriaticum* CCMP2467 across pH treatments. Letters indicate overall similarities (e.g., AA) or differences (e.g., AB) between treatments as determined by SNK *post hoc* tests.

Our transcriptomic analyses showed enrichment of genes and processes involved in photosynthesis and carbon acquisition mechanisms, with most of the DEGs being significantly up-regulated. To test whether these transcriptomic changes were evident on the physiological level, we measured photosynthetic efficiency of the symbiont using Pulse-Amplitude-Modulation fluorometry. Seawater pH had a significant effect (ANOVA *F* = 7.503, *p* = 0.005) on the photo-physiological performance (*F’/Fm’*) of *S. microadriaticum* CCMP2467 (Figure 6). Specifically, lower *F’/Fm’* values were observed in colonies from the more acidic treatments (pH 7.2 and 7.4) compared to the control (pH 8.0) whereas no significant differences were detected for coral fragments from pH 7.8. Interestingly, however, is that photochemical yields dropped significantly from pH 7.8 to pH 7.4 (mean value ± SD, from 0.5 ± 0.02 to 0.4 ± 0.02) but remained stable between pH 7.4 and pH 7.2 (mean ± SD, 0.4 ± 0.02 and 0.4 ± 0.03, respectively).

## Discussion

### Responses of Coral Holobionts to OA Vary Greatly

Transcriptome sequencing has become a powerful tool for understanding the mechanistic underpinnings of coral resilience to environmental change by allowing the identification of genes and/or pathways that are specifically associated with stress responses (reviewed in Cziesielski et al., 2019; Strader et al., 2020). However, most studies have focused on the coral host and it was not until recently that changes in gene expression following exposure to high temperature and/or CO_2_ levels have also been examined in their algal symbionts (Kenkel and Matz, 2016; González-Pech et al., 2017; Davies et al., 2018; Kenkel et al., 2018; Rivest et al., 2018), thus providing a more complete view of the holobiont responses. Indeed, both partners exhibit differential transcriptomic changes; some studies have shown that the host elicits a stronger response (up to five times) (Kaniewska et al., 2015; Kenkel and Matz, 2016; Davies et al., 2018) whereas others have found greater effects on the symbiont (Kenkel et al., 2018; Rivest et al., 2018). The magnitude of the response to different stressors also varies greatly. Generally speaking, warming, alone or in combination with acidification, has a larger effect on gene expression than just OA (Rocker et al., 2015; Davies et al., 2016, 2018; Rivest et al., 2018). For example, Davies et al. (2016) found almost 25% of the holobiont meta-transcriptome to be differentially expressed in response to temperature stress whilst less than 2% was attributed to elevated *p*CO_2_. Similarly, minimal (or no) transcriptomic responses to low seawater pH (close to RCP8.5 scenarios) have also been reported (Vidal-Dupiol et al., 2013; Kaniewska et al., 2015; González-Pech et al., 2017). Hence, it is not surprising that in our study, changes in expression only accounted for a small fraction of the transcriptome of both symbiont and host. Furthermore, the transcriptional responses to acute stress and chronic stress differ substantially (reviewed in Strader et al., 2020). An initial response to acute stress generally invokes greater changes in gene expression as a mean to regain homeostasis, whereas long-term chronic stress exposure implies that acclimation has already occurred and transcriptional changes are limited to processes necessary to maintain homeostasis (via up-/down-regulation of certain genes, for example).

Numerous studies have reported mixed findings on the effects of high *p*CO_2_ on the photo-physiology of symbiotic cnidarians. For example, acidification stress has been shown to increase productivity, symbiont density and photochemical efficiency in non-calcifying anthozoans (Suggett et al., 2012; Towanda and Thuesen, 2012; Jarrold et al., 2013; Gibbin and Davy, 2014; Horwitz et al., 2015; Klein et al., 2017) but the opposite in corals (Anthony et al., 2008; Crawley et al., 2010; Edmunds, 2012; Kaniewska et al., 2012; Zhou et al., 2016), and in fewer cases, no significant effects have been observed (Wall et al., 2014; Tambutté et al., 2015; Davies et al., 2018). Certainly, the intertwined interaction between photosynthesis and calcification (Furla et al., 2000) might be key for determining how and to what extent OA will affect corals. Indeed, while OA has been shown to be detrimental to adult coral colonies (see above), contrasting responses have been observed in young recruits and larvae. For example, Jiang et al. (2019, 2020) found a positive photochemical response to OA along with enhanced symbiont CCMs in *Pocillopora* recruits and greater productivity in larvae. Finally, parabolic responses of photosynthetic processes to acidification stress have also been documented (Crawley et al., 2010; Castillo et al., 2014). Different *p*CO_2_ levels and the diverse CMMs of symbionts (Buxton et al., 2009; Brading et al., 2011) may contribute to these variable and contrasting responses. Finally, responses to acidification stress are not only symbiont strain-specific (Buxton et al., 2009; Brading et al., 2011) but may also differ depending on whether they are free-living or *in hospite* different hosts (see above).

### Coral Symbionts Acclimate to OA by Fine-Tunning Gene Expression and Photo-Physiology

This study provides novel insights into coral acclimation to OA by integrating the findings presented here with previously published data (Tambutté et al., 2015; Liew et al., 2018) that support our interpretation of the results. Although we did not find significant differences in expression levels across treatments for the symbiont, there were still a number of genes that were significantly differentially expressed and biologically meaningful. Most of the changes in gene expression we observed here were associated with homeostasis and regulation of metabolic functions; yet we were particularly interested in those involved in photosynthesis and carbon-concentrating mechanisms (CCMs). These processes are essential for holobiont functioning but also different from the other biological processes in that they could potentially benefit from increased *p*CO_2_. Over-expression of genes encoding for different cellular components critical for the photosynthetic machinery was common in both extreme treatments (pH 7.2 and pH 7.4); specifically, polypeptide subunits of photosystems, light-harvesting and oxygen-evolving complex proteins responsible for enhancing recruitment and functioning of PS II and several enzymes necessary for photosynthesis. Enrichment of H^+^ transporter genes were also observed; in particular, a vacuolar H^+^ ATPase (VHA) proton-pump essential to promote photosynthesis as it catalyzes ATP hydrolysis and lowers pH in the symbiosome (Barott et al., 2015). Indeed, inhibition of this transmembrane protein can result in reduced photosynthetic activity (Barott et al., 2015). Moreover, up-regulation of proton-pumps from the symbiont but also bicarbonate transporter-like proteins from the coral in response to acidification stress suggests that the holobiont might be trying to compensate and/or maintain the internal pH gradient necessary to support nutrient exchange between partners (Barott et al., 2015). Bicarbonate transporters are, in turn, important for the host as these not only play a role in biomineralization but also in homeostatic control of metabolic processes (Zoccola et al., 2015). Previous evidence even suggests that these transporters may play an important role in coral resilience to acidification (Zoccola et al., 2015), thus contributing to the notion that changes in their expression might be indeed an acclimation mechanism of the host to different water chemistries.

Neither host carbonic anhydrases nor solute carrier (specifically SLC4 and SLC26) genes were differentially expressed in any of the treatments, which was initially surprising because these have been shown to be significantly up-regulated in corals subjected to only few weeks of acidification stress (Vidal-Dupiol et al., 2013) (but see above for the effect of exposure time in expression levels). Both play a major role in CCMs; carbonic anhydrases move carbon from the seawater environment across multiple layers into the symbiont cell whilst SLCs are responsible for transporting HCO_3_^–^ and other ions, however, the main role of these specific transporters might be the provision of bicarbonate to the calcification process (Zoccola et al., 2015). Significant enrichment of symbiont-containing vacuole membrane (which here may refer to the host-derived symbiosome) further highlights the effect of elevated *p*CO_2_ on the holobiont CCMs. Acidification of this intracellular compartment is essential for promoting photosynthesis (Barott et al., 2015) and damage and/or disruption of its cellular components and functions could have a negative impact on host-symbiont communication and metabolic exchange. Indeed, a gene encoding for guanylate-binding proteins, previously associated with resistance to intracellular pathogenic microbes (Haldar et al., 2013), was significantly over-expressed. This might suggest the host’s immune response is compromised and secretion of this protein increases as a compensation measure.

Despite previous evidence for beneficial effects from elevated *p*CO_2_, often reflected by enhanced productivity (Strahl et al., 2015; Biscéré et al., 2019) and here seen as an enrichment of processes related to photosynthesis and carbon acquisition, our findings suggest that this might be instead an acclimation response of the holobiont to OA. For example, although here we observed significant declines in the operating efficiency of PS II (*F’/Fm’*), gross photosynthesis rates (as previously reported in Tambutté et al., 2015 for the same coral genet used in this study) have been noted to remain unchanged. Discrepancy between these two measurements has been observed before (Briggs and Carpenter, 2019); multiple aspects of photo-physiology may indeed respond differently to acidification stress with potentially contrasting consequences for photosynthetic performance. The expression of specific proteins and/or maintenance of enzymes related to photo-physiology may be altered, and though this might not influence photosynthesis rates, it could have a significant effect at the cellular level. Protein turnover is particularly important for photosynthesis as it allows repairing damage to the PS II (Han, 2002; Briggs and Carpenter, 2019), thus changes in protein metabolism can eventually lead to a reduction in photochemical efficiency. Indeed, here we observed changes in expression of protein metabolism related functions (most of them depleted) such as protein transport (GO:0015031), intracellular protein transport (GO:0006886), ubiquitin protein ligase binding (GO:0031625) and protein autoubiquitination (GO:0051865), among others.

Other physiological measurements like respiration rates, symbiont densities and protein content were not affected either (also reported in Tambutté et al., 2015). Thus, suggesting that although the overall performance of the holobiont is not (seriously) compromised, the ability of this particular host-symbiont association to maintain carbon fluxes might be affected by OA. The latter because photosynthesis and respiration depend on the availability of dissolved inorganic carbon (Furla et al., 2000; Nakamura et al., 2013) so that more substrate should theoretically enhance both processes; higher CO_2_ fixation during photosynthesis results in increased oxygen production and thus stimulating respiration (Reece et al., 2015). Though this only holds true if the process is CO_2_-limited; otherwise, an increase in CO_2_ will not affect oxygen fluxes as the system is already working at maximum capacity. Another possibility to consider is that CO_2_ might not be efficiently acquired. This is surprising, however, since *S. microadriaticum* CCMP2467 in particular is known to have many more bicarbonate transporter domains than other symbiotic dinoflagellates (Aranda et al., 2016). As such, one would assume that it is more efficient in mobilizing HCO_3_^–^ across membranes. Nonetheless, it could also be that CCMs in this symbiont are mainly supported by the uptake of CO_2_ and not HCO_3_^–^, as it is the case for *S. necroappetens* (formally known as *Symbiodinium* A13; LaJeunesse et al., 2015) (Brading et al., 2013), for example. Thus, having more bicarbonate transporters might not necessarily improve carbon transport for photosynthesis. Further, CCMs are active, energy-consuming processes (Leggat et al., 1999) thus if the host is challenged in any way (see above), these can become less efficient and therefore limiting, independent of the symbionts ability to assimilate CO_2_.

### Methodological Considerations

We are cautious when interpreting the effect of pH on gene expression (i.e., PERMANOVA tests to assess differences between treatments) as low statistical power (because of low sample size) can negatively affect the likelihood that a statistically significant finding actually reflects a true effect. Ideally, more samples (i.e., coral nubbins) from different genotypes in each condition should be examined. Yet, this dataset was already published by Liew et al. (2018), who investigated the epigenetic mechanisms underlying OA stress responses (and thus used samples with the same genetic background in order to compare across treatments). Here, we simply provide initial transcriptomic observations of a coral endosymbiont to OA with respect to photosynthesis. This study adds to the growing body of work investigating how different marine organisms respond to OA. Similar efforts on this and other coral-Symbiodiniaceae pairings will eventually reveal the extent at which our findings can be generalized.

Finally, it is also worth noting that, as well as with other environmental factors (e.g., temperature and nutrients), light availability plays an important role in regulating calcification and photosynthetic processes under elevated *p*CO_2_ (Suggett et al., 2013). Specifically, it has been shown that corals under low light growth conditions exhibit greater OA-induced calcification loss and lower gross photosynthesis rates. Since the light intensity used in this study (170 μmol photons/m^2^s) is similar to the one (100 μmol photons/m^2^s) tested by Suggett et al. (2013), it is fair to consider the possibility that even if *Symbiodinium* has the capacity to use CO_2_ at lower pH, photosynthesis might have not been increased because of the low light. Under low light conditions, photo-acclimation processes that facilitate light harvesting also require protein production and maintenance which, as discussed above, is negatively affected by OA (Briggs and Carpenter, 2019). This might explain the increasingly detrimental effects on photochemical efficiency as pH decreases. OA studies should then better account for the potential moderating role of light on elevated *p*CO_2_ as together they clearly alter the energetic budgets and resource allocation among photosynthetic processes (Suggett et al., 2013; Briggs and Carpenter, 2019).

## Conclusion

Here, we present an overview of the *in hospite* transcriptomic response of a common coral symbiont exposed to different seawater pH conditions. This study together with Kenkel et al. (2018) are, to our knowledge, the only ones examining changes in expression patterns under long-term CO_2_ acidification stress. Their contrasting findings, as they did not show differential expression of any gene related to photosynthesis nor carbon acquisition mechanisms, further highlight the importance of investigating different host-symbiont pairings to better understand the effects of OA on corals. Certainly, host and symbiont genetics (and the interaction between both) play a major role in gene expression in response to stress (Parkinson et al., 2015; Cziesielski et al., 2019; Strader et al., 2020) such that conclusions based on this experiment may only be applicable to the host-symbiont combination in question and care should be taken in extrapolating this response to other coral-Symbiodiniaceae assemblages. In summary, our data suggest that, despite the existing hypothesis that elevated CO_2_ benefits photosynthetic organisms, here, up-regulation of genes involved in photosynthesis processes might be an acclimation response to OA stress experienced by the host (and thus (indirectly) by the symbiont) and not a beneficial effect. Indeed, we show a significant drop in photochemical yields with increasing *p*CO_2_, which in turn demonstrates the extent of symbiont photo-acclimation that operates in response to a changing environment (reviewed in Nitschke et al., 2018). The implications of these changes under extended acidification stress and the effect this may have on symbiotic interactions is complex and thus warrants further study, especially since responses vary greatly among different host-symbiont combinations.

## Data Availability Statement

All sequencing data from this study has been deposited in NCBI (https://www.ncbi.nlm.nih.gov/bioproject/PRJNA386774; Liew et al., 2018).

## Author Contributions

MA and GC conceived and coordinated this project. MA, YL, AV, ET, DZ, and ST provided tools, reagents, and data. AV and ET performed the experiments. MH and GC analyzed expression data. MH wrote the manuscript with help from MA. All authors read and approved the final manuscript.

## Conflict of Interest

The authors declare that the research was conducted in the absence of any commercial or financial relationships that could be construed as a potential conflict of interest.

## Footnotes

1 http://www.reefgenomics.org

2 https://github.com/lyijin/topGO_pipeline

3 www.genome.jp/tools/kaas/

4 https://github.com/lyijin/common/blob/master/kegg_backgrounds/
